# Round the Hospitals

**Published:** 1920-11-27

**Authors:** 


### ROUND THE HOSPITALS.

The Duke of York made a stirring speech on the occasion of the Dinner at the Connaught Rooms last week in aid of the Queen's Hospital for Children, and it met with a generous response from the distinguished company gathered on the occasion. The Queen's Hospital is situated in a district not very favourable for the healthy development of child life, and unless its opportunities for healing can be kept uncurtailed an immense mass of unrelieved suffering, a deplorable wreckage of still curable lives, must result. The expenses have more than doubled. The premises, and particularly the out-patient department, call imperatively for enlargement. The appeal is for £25,000 to carry out alterations and meet current expenses. The Bishop of London, in a moving speech, declared he had himself carried in many of the little patients to the hospital, and assured his hearers from personal knowledge that many of these invalid children had no bed only a heap of rags, no nurse but an over-worked mother, and that to transfer such children to the Queen's Hospital was like moving them from hell to heaven. "They were saving life by handfuls." The Secretary, Mr. T. Glenton Kerr, was able to announce a subscription list of £9,000 already. Much amusement was caused by the donation of £555 from "The Brotherhood of the Cheerful Sparrows."

Princess Beatrice and the Duchess of Albany were present last week at a meeting of the League of Remembrance, which perpetuates the memory of the War Hospital Supply Department Movement. It is now interesting itself on behalf of the Red Cross Hospital Library, which circulates books and magazines to the British naval, military, and civil hospitals at home and abroad. Many charming anecdotes were recounted by Miss Beatrice Harraden and Mr. Pett Ridge to illustrate the satisfaction imparted to patients by books of almost every description. One curious petition, it was related, came from a matron at Assouan for an old A.B.C. guide for a patient, whose only solace was to work out the return fare to different places at home. The value of getting fresh supplies of literature for the patients at stated intervals is incalculable. Hospitals cannot store a great quantity of books. The shelves on which the patients' volumes repose have a way of growing steadily shabbier, and, unless replenished from time to time, they are liable to be summarily cleared off by tidy sisters. The hospital library is, in fact, doing a good work, and the business of collecting for it ought to be regularly undertaken by hospital visitors. Its headquarters are at 1 Marlborough Gate, W. 2.

The Middlesex Hospital is on the eve of inaugurating a system of payment for patients. In-patients are to pay according to means. The Earl of Athlone, Chairman of the Board of Management, who presided at the recent meeting called to decide on this step, gave startling figures illustrating the difficulties of carrying on under the purely eleemosynary system. In protesting that the voluntary system was not dead, he spoke with enthusiasm of the services rendered to the public by the hospitals in the fostering of medical science, which, as it stands to-day, was he declared, "the offspring of the hospitals." It is seldom indeed, we fear, that those who experience the great comfort of good nursing in their own homes are brought to realise the debt they owe to the hospitals for training nursing sisters. Yet this part of their work becomes more costly day by day. It should be brought into evidence more than it is, in reports, in speeches, and appeals. The trained nursing service, of which rich and poor enjoy the fruits in a hundred different directions, is truly "the offspring of the hospitals." The public should not be allowed to forget this.

Most nurses discover after but a short experience of health work that ailing men, women, and children respond to no form of treatment so quickly as to one thoroughly satisfactory meal a day, rich in vitamines, well cooked, and eaten in comfortable surroundings. Hence the value in poor and crowded quarters of public invalid kitchens, such as the one opened at 12 Windsor Street, Islington, last week. The Queen is the patron of the "Invalid Kitchens of London," and the Duchess of Somerset, unfortunately absent on this occasion owing to an attack of influenza, is the President. Lady Helen Cassel explained that nursing mothers and convalescents recently discharged from hospital were among those who were to benefit by the new centre. Only £1,000 is needed to ensure the success of the venture and we trust it will speedily be forthcoming. It will be a powerful auxiliary to the nursing work of the district.

Miss Gullan, sister-tutor at St. Thomas's, has produced a very valuable handbook for the use of nurses in her "Theory and Practice of Nursing" (H. K. Lewis & Co., price 12s. 6d.). The book is a summary of the instruction given to nurses in training in the Nightingale School. It is adapted to continue the teaching begun in a preliminary training-school, and is meant to supplement ward instruction, so that much purely routine work is omitted. Nurses are recommended to use the volume for making a systematic revision every three or six months during training. Blank leaves are inserted, on which brief notes of clinical and practical experience can be entered. It is the nearest approach to a truly scientific exposition of nursing which we have encountered. If to instruct means, as the Latin derivation implies, "to draw up in order," this is the perfect mode of instruction. It deals with a mass of hitherto unclassified data, and presents them in a co-ordinated form in which memory and reasoning powers interact, and the nurse in training is encouraged to regard herself as a student with brains of her own. Wherever the volume be opened the reader comes upon evidence of clear thinking and compact treatment. It is the

**ROUND THE HOSPITALS**—*(continued).*

book for which matrons and sister-tutors have long been sighing, and we predict a long future for it.

Nurses should not fail to visit the galleries of Messrs. Agnew in Old Bond Street at their earliest moment of leisure, for there a highly interesting collection of pictures is on view. The entire proceeds of the exhibition, which remains open till Christmas, are to be devoted without any deduction for expenses to the Nation's Fund for Nurses. We trace this generous offer to the publicity afforded to the Fund through the *Daily Telegraph*, of which the fruits are still observable in more than one direction.

The Children's Jewel Fund, which reaped so rich a harvest for the sick and suffering in so pleasant a manner, is in a position to make more grants. It is proposed to establish two memorials, one of which is the endowment of a maternity ward at Queen Mary's Hospital for the East End. The other is to take the form of a centre for treating infants suffering from mal-nutrition. The sum of £3,500 has been set apart for each of these schemes.

A distressing case of suicide occurred last week at the Parc Gwyllt Asylum, Bridgend, Glamorgan, and at the inquest held subsequently one of the nursing staff, Nurse Thomas, had a rather remarkable story to tell. She said that the patient in question, a young woman, formerly a schoolmistress, who was admitted in June last suffering from depression, was with her in the kitchen when she had occasion to go upstairs to get a bottle of carbolic acid from a locked cupboard in a day-room for some dressings. She had unlocked the cupboard and taken the bottle in her hand before she was aware of the fact that the patient had stealthily followed her upstairs and was standing at her side. Like a flash the young woman seized the bottle and dashed away, holding it to her lips, the nurse in swift pursuit. Unfortunately Nurse Thomas tripped in the door of another room, or she might have caught the patient in time to save her life. As it was, the latter had drunk two or three tablespoonfuls of the acid, and though the institution medical officer was summoned at once and applied the stomach pump, continuing his efforts for nearly a couple of hours, the patient succumbed to the poisonous dose. A strange feature of the case was that the young woman had not been regarded as of suicidal tendency, though it came out at the inquest that before her admission to the institution she had thrice tried to drown herself. A *post-mortem* examination, moreover, revealed the presence in her stomach of a quantity of twisted hairpins, washers, needles and a screw. The coroner said that it was obvious Nurse Thomas was not to blame in the least degree.

The Laundry Exhibition at the Royal Agricultural Hall has been attracting a great deal of attention from institution managers. It deals with every detail of laundering, and marks a great advance upon the last, held the year before the war. The machinery now obtainable makes it possible to deal with soiled linen rapidly without risk of deterioration, with the minimum of labour, and at a low cost for fuel. The direction of laundries forms an interesting and well-paid occupation for women, and is a very suitable one for nurses who for any reason are abandoning their own career. It demands, in addition to a knowledge of the technical parts of the business, which can be readily acquired, just that trained talent for superintending others which nursing develops. There is no excuse in the present day for hospitals to give out their laundry to be executed under conditions which they cannot control, for even the smallest institution can instal its own plant, under the skilled advice available.

The concert organised by the nursing staff, under the direction of Sisters Hill, Forbes, Smith, and Aughton, in aid of the Nurses' Home for the Great Northern Central Hospital proved a great success. There was an excellent and varied programme of songs and recitations, and Miss Gertrude Jennings' farce "In the Cellar" was greeted with much applause and laughter. By kind permission of Mr. Cosmo Hamilton, his sketch "In the Haymarket" was performed.

The Central Midwives Board for Scotland held their examinations on November 1 and 2 simultaneously in Edinburgh, Glasgow, Dundee and Aberdeen. The number of successful candidates has grown apace. The total is 138, including pupils at the following institutions:—Royal Maternity Hospital, Rottenrow, Glasgow, 50; Royal Maternity Hospital, Edinburgh, 23; Cottage Nurses' Training Home, Govan, 20; the Maternity Hospital, Dundee, 13; Queen Victoria's Jubilee Institute, Edinburgh, 7; the Maternity Hospital, Aberdeen, 6; and other recognised institutions, 19.

Walthamstow and Leyton District Churches and Charities have lost a valuable worker and friend through the recent death of Hariet Helen Ruck-Keene, daughter of the late Archdeacon Long. Upon the death of her husband in Italy in 1900 Mrs. Keene decided—although then seventy years of age—to devote the rest of her life and energies to Christian and social work, and having secured Forest Lodge, Leyton, a fine old family residence, opened it as a ladies' settlement to act as a centre for those willing to undertake social and religious work in the district. As a supporter of hospital and dispensary effort Mrs. Keene was always to the fore, while perhaps in this connection her greatest help was given in 1918; for when on account of failing strength her work at Forest Lodge had to be given up, she offered this well-equipped and now recently enlarged building to the Marie Celeste Society of the London Hospital as a convalescent home for women and children, if they would undertake the cost of maintenance. The offer was gladly accepted and valuable work has been the result, as a floral tribute testified, which was sent to the funeral in the name of some 400 children who had passed through the wards during the last two years.

